# Supplementary material for: ISG15 as a Potent Immune Adjuvant in MVA-Based Vaccines Against Zika Virus and SARS-CoV-2
Source: Vaccines (Basel). 2025 Jun 27;13(7):696. doi: 10.3390/vaccines13070696 (PMC12300054; doi:10.3390/vaccines13070696)
Supplement: Supplementary file 1 [file vaccines-13-00696-s001.zip › vaccines-3703285-supplementary.pdf]

A

```
mISG15   1    MAWDLKVKMLGGNDFLVSVTNSMTVSELKKQIAQKIGVPAFQQRLA-HQTAV-LQDGLTL   58
hISG15   1    MGWDLTVKMLAGNEFQVSLSSSMSVSELKAQITQKIGVHAFQQRLAVHPSGVALQDRVPL   60

mISG15   59   SSLGLGPSSTVMLVVQNCSEPLSILVRNERGHSNIYEVFLTQTVDTLKKKVSQREQVHED   118
hISG15   61   ASQGLGPGSTVLLVVDKCDEPLSILVRNNKGRSSTYEVRLTQTVAHLKQQVSGLEGVQDD   120

mISG15   119  QFWLSFEGRPMEDKELLGEYGLKPQCTVIKHLRLRGGG   156
hISG15   121  LFWLTFEGKPLEDQLPLGEYGLKPLSTVFMNLRLRGGG   158
```

B

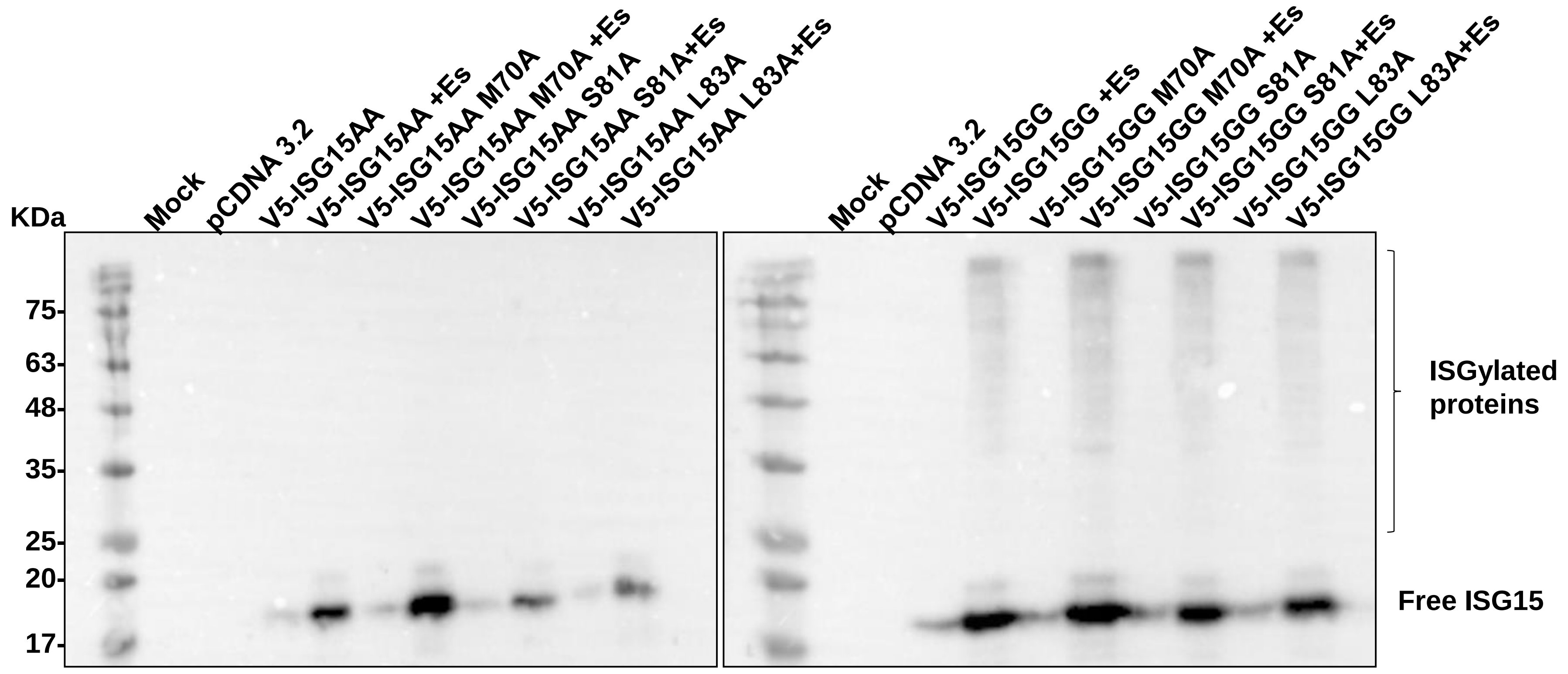

Supplementary Figure S1. Characterization of Murine ISG15 Mutants: Sequence Alignment and *In Vitro* Expression Analysis. (a) Alignment of murine and human ISG15 sequences. Generated mutants and their associated functions are indicated. (b) In vitro expression of ISG15. Expression of ISG15GG and ISG15AA proteins in cell extracts from 293T cells transfected with pcDNA3, pcDNA3-V5mISG15-GG, pcDNA3-V5mISG15-AA, and their respective mutated versions at 48 hours post-transfection. Western blot analysis was performed using a hamster monoclonal anti-ISG15 antibody to detect ISG15 proteins. The positions of free ISG15 and ISGylated proteins are indicated on the right.

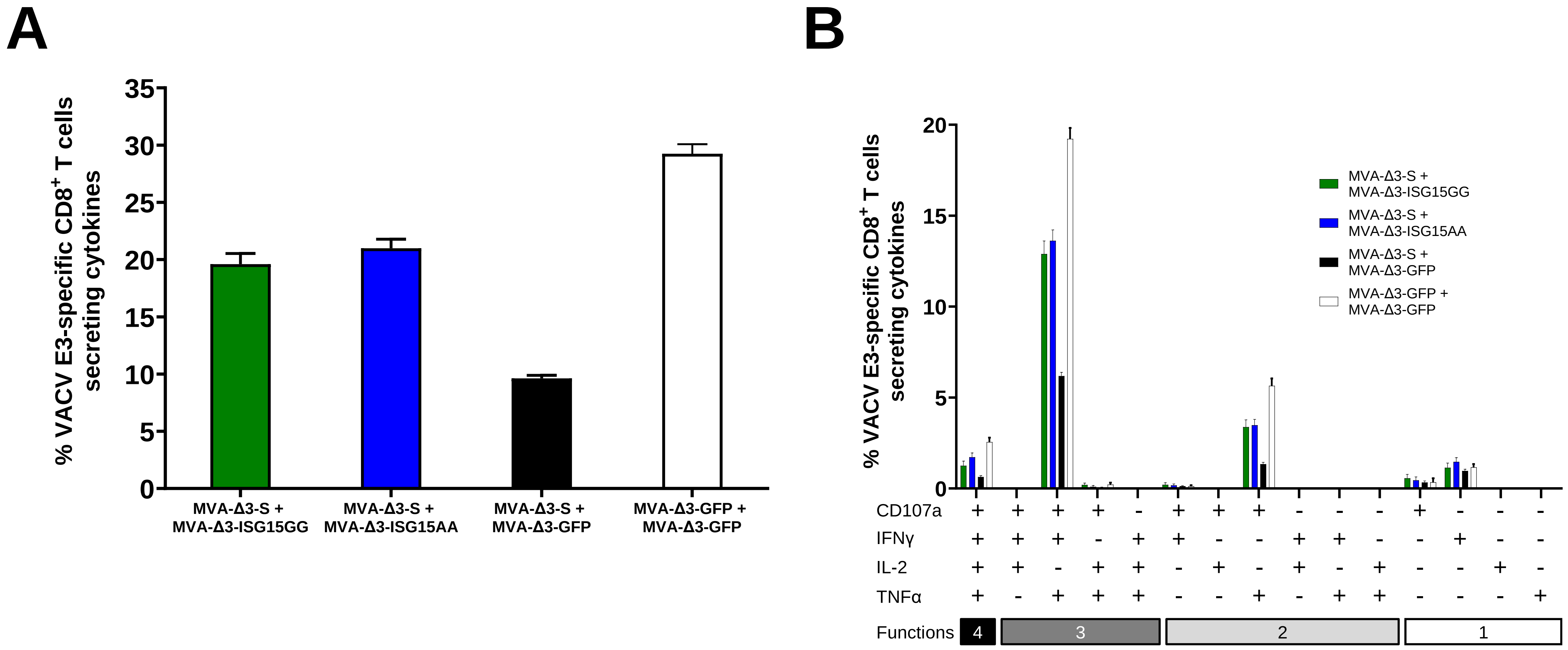

Supplementary Figure S2. Vector-specific CD8 T-cell immunogenicity following homologous MVA prime/boost regimens in the presence of ISG15. Female C57BL/6 mice (n=5/group) were co-immunized at week 0 and 2 with MVA-Δ3-S + MVA-Δ3-GFP, MVA-Δ3-ISG15GG or MVA-Δ3-ISG15AA. At 10 days after the boost, spleens were obtained and VACV E3-specific responses were evaluated by ICS after stimulation of splenocytes with a VACV E3 peptide. (**A**) Magnitude of VACV E3-specific CD8 T cells. The values represent the sum of the percentages mean and SD of T cells producing CD107a and/or IFN-γ and/or TNF-α and/or IL-2 against the VACV E3 peptide. (**B**) Polyfunctional profile (based on expression of selected markers CD107a, IFNγ, TNFα and IL-2) of VACV E3-specific CD8 T cell immune responses. Percentages mean and SD of VACV E3-specific CD8 T cells producing 4, 3, 2 or 1 cytokine. The response profiles are shown on the x axis, and the percentages of T cells for each of the vaccination groups are shown on the y axis.
